# Response of Leaf Traits and Photosynthetic Fluorescence Characteristics of *Fraxinus malacophylla* Seedlings to Rainfall Patterns During Dry and Rainy Seasons in Southwestern China

**DOI:** 10.1002/ece3.70363

**Published:** 2024-10-15

**Authors:** Huiping Zeng, Xiaofei Cha, Lijuan Sun, Huanxian Guo, Shaojie Zheng, Xingze Li, Qiong Dong

**Affiliations:** ^1^ College of Forestry Southwest Forestry University Kunming Yunnan China; ^2^ Southwest Mountain Forest Resources Conservation and Utilization of the Ministry of Education Kunming Yunnan China; ^3^ Nujiang Prefecture Forestry and Grassland Bureau Nujiang Yunnan China

**Keywords:** dry and rainy seasons, *Fraxinus malacophylla*, leaf traits, photosynthetic fluorescence characteristics, rainfall patterns

## Abstract

Global climate change has led to a shift in rainfall patterns. And as water is an essential ingredient for plant photosynthesis, shifts in rainfall patterns will inevitably affect plant growth. This study was conducted in Kunming, southwest China. In this study, the response of leaf traits and photosynthetic fluorescence properties of *Fraxinus malacophylla* seedlings to rainfall patterns during the dry and rainy seasons was investigated using a natural rainfall interval of 5 days (T) and an extended rainfall interval of 10 days (T_+_) as rainfall interval treatments and a monthly average rainfall as a control (W), with the corresponding rainfall treatments of a 40% increase in rainfall (W_+_) and a 40% decrease in rainfall (W_−_). The results showed that Pn, Gs, and Tr basically all tended to increase and then decrease with increasing rainfall in the dry season and generally reached the highest under the W treatment; Pn, Gs, Ci, and Tr mostly remained high at 5 days relative to 10 days; PI was overall higher under the W treatment throughout the dry season. Extending the rainfall interval at the beginning of the rainy season significantly reduced Fm; throughout the rainy season, Gs, Ci, and Tr basically showed a decreasing trend with increasing rainfall, reaching the highest under the W‐treatment and mostly higher at 5 days than at 10 days. These results suggest that natural rainfall intervals and natural rainfall amounts are more favorable to the growth of *Fraxinus malacophylla* seedlings in the dry season; reduced rainfall and multiple rainfalls in the rainy season tend to promote photosynthesis in *Fraxinus malacophylla*. This study reflects the different survival strategies of *Fraxinus malacophylla* under different rainfall patterns, as well as provides a theoretical basis for understanding how *Fraxinus malacophylla* can grow better under rainfall variability and for future management.

## Graphical abstract

Global climate change has led to a shift in rainfall patterns. And as water is an essential ingredient for plant photosynthesis, shifts in rainfall patterns will inevitably affect plant growth. In this study, the response of leaf traits and photosynthetic fluorescence properties of *Fraxinus malacophylla* seedlings to rainfall patterns during the dry and rainy seasons was investigated using a natural rainfall duration of 5 days (T) and an extended rainfall duration of 10 days (T+) as rainfall intervals; and a monthly average rainfall as control (W), with the corresponding rainfall treatments of a 40% increase in rainfall (W_+_) and a 40% decrease in rainfall (W_−_).

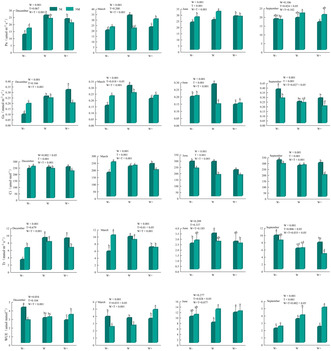

## 1 Introduction

In natural ecosystems, the amount and frequency of natural rainfall are important factors influencing the distribution of plants in terrestrial ecosystems, the formation of biodiversity, and the realization of ecosystem functions (Zhang 2022). In a situation of dramatic global climate change, rainfall patterns (rainfall amount, rainfall interval, and seasonal concentration of rainfall) will cause plants to experience transient cycles of soil moisture deficit (Hu et al. 2022). In turn, soil moisture affects many processes and life cycles, such as soil pH, diffusion of gases, nutrient availability, microbial migration, diffusion of compounds between the cells of an organism and the environment, and mineralization rates (Furtak and Wolińska 2023). These factors in turn affect soil fertility and productivity, which in turn affect plant growth, development, and metabolism. According to meteorological data analyses and global climate model projections, the arid and semi‐arid regions of central Asia are experiencing significant changes in rainfall, with an increase in the amount of single rainfall, an increase in rainfall intensity (Zhang, Wang, et al. 2023; Zhang, Yang, et al. 2023), and a prolongation of drought (Anson 2008). Drought is one of the main factors limiting plant growth in these areas; it impedes respiration, stomatal movement, and photosynthesis, which impacts the physiological and metabolic processes as well as the growth and development of plants (Hartmann and Trumbore 2016; Sainju, Lenssen, and Ghimire 2017). Therefore, improving the efficiency of photosynthesis and promoting plant growth and development under drought conditions has increasingly become an important research topic for researchers around the world (Hu, Zhang, and Guo 2023). However, current studies on plants under rainfall variability mainly include seed germination (Zhang 2021), seedling biomass (Bai et al. 2017), root morphology (Shan et al. 2017), in addition to leaf plasticity (Mendes et al. 2022), gas exchange, and leaf anatomy (Marcelo et al. 2024), while studies on plant leaf traits and photosynthetic fluorescence characteristics have rarely been reported, and studies on the effects of abiotic stress rainfall variability on plants are mainly focused on an individual month or dry season, while few studies are carried out under different months or different seasons. Thus, studying the changes in leaf traits and photosynthetic fluorescence parameters of *Fraxinus malacophylla* seedlings under different rainfall pattern treatments in the dry and rainy seasons will be helpful in revealing how to promote plant growth in the dry season and, at the same time, providing some references for how to promote plant growth in the rainy season.

Leaf traits mainly include leaf area, specific leaf area, leaf shape index, leaf water content, etc., which can respond to the growth of plants under specific environmental conditions and reveal the physiological response mechanisms of plants under environmental changes (Hamid et al. 2014). Since plant leaf traits are relatively macroscopic and sensitive to climate change, they are often used as observational indicators to study the adaptive responses of relevant plants to precipitation changes (He et al. 2024). For example, precipitation changes significantly affected the leaf traits of plant communities and *Leymus chinensis*; short‐term extreme drought had a significant effect on the leaf area of typical grassland communities and *Leymus chinensis*; as the amount of precipitation decreased, the leaf area of communities and *Leymus chinensis* decreased while the specific leaf area increased; and typical grassland plant communities adapted to precipitation changes through dominant plant changes in major leaf traits to adapt to changes in precipitation, including adaptation to short‐term extreme drought through changes in leaf area (Yue et al. 2018). Specific leaf area (SLA), defined as the ratio of leaf area to leaf dry mass, is commonly used as a parameter in ecosystem modeling and as an indicator of the potential growth rate, which can reflect the potential growth rate of organs associated with resource capture from leaves (Sabine and Folkard 2019). Research has found that the specific leaf area of plants is sensitive to changes in the external environment and changes continuously with the environment (Freschet, Swart, and Cornelissen 2015). In a study of *Larix decidua* Mill. (Fellner, Dirnberger, and Sterba 2016), it was shown that low water uptake by plants reduces the specific leaf area of the plant. Whereas a study on young trees of three *Adansonia digitata* species in Madagascar, Africa (Randriamanana et al. 2012), showed that the effect of drought on specific leaf areas was not significant. Compared to regions with abundant precipitation, arid and semi‐arid regions have smaller plant leaf areas. This is primarily because the reduced plant leaf area effectively reduces water loss from transpiration, allowing a limited amount of water to be used for the parts of the plant that are essential for survival (Huang, Liang, and Hang 2008). This indicates that environmental changes can affect plant leaf traits and also reflects the close relationship between plant leaf traits and individual plant behavior and function (Mao et al. 2012). Therefore, understanding the changes in leaf traits of *Fraxinus malacophylla* seedlings under different precipitation environments will help to reveal the intrinsic mechanism of plant response to environmental changes and the relationship between water and plants, as well as linking to photosynthesis in plants and determining the strength of photosynthesis occurring.

Photosynthesis is the most basic requirement for the plant body to achieve growth, development, and metabolism, and it is also one of the physiological activities to which plants are most vulnerable to abiotic stresses (Sajad et al. 2017). Leaf photosynthetic parameters have great potential for use in contemporary forest and crop breeding to increase biomass and yield, and changes in leaf photosynthetic parameters of forest trees reflect their ecological strategies and adaptations in response to environmental changes (Qu et al. 2017). Moisture is the basis for a series of physiological and biochemical reactions in plants; therefore, the photosynthetic parameters of forest tree leaves are altered when precipitation is changed (Sardans and Peñuelas 2012). For example, a study on three *Quercus ilex* (Martin‐StPaul et al. 2012) from sites with different annual rainfall showed no significant difference in stomatal conductance at sites with abundant rainfall; however, populations from the wettest areas showed a greater decrease in stomatal conductance with decreasing leaf water potential than those from the driest areas, and higher growth rates and plant heights were observed at the wettest sites. Studies on the effect of root water uptake on the stability of plant slopes found that an increase in water uptake by the plant root system increased the transpiration rate of the plant, which was more favorable to plant slopes (Cheng et al. 2024). However, the study on the effect of rainfall variation on the photosynthesis of *Fraxinus mandshurica* (Zhan et al. 2012) found that the effect of rainfall variation on growth and photosynthesis of *Fraxinus mandshurica* seedlings was not significant. Currently, the results of studies on the effects of changes in rainfall patterns on photosynthetic parameters of forest leaves are inconsistent and are related to individual development (Myers and Kitajima 2007) and climatic environment (Xu et al. 2020), in addition to depending on tree species. Therefore, it is particularly important to study the changes in photosynthetic parameters in the leaves of *Fraxinus malacophylla* seedlings in a climatic environment with changing rainfall patterns.

Chlorophyll fluorescence parameters reflect the photosynthetic physiological properties of plants under environmental stress and are often used as photosystem probes of photosynthesis (Wang, Li, et al. 2023; Wang, Zhang, et al. 2023). Reduced water will make plant photosynthesis efficiency decrease, leading to an increase in excess light energy and resulting in photoinhibition, thus damaging the photosystem of the photosynthetic apparatus (PS II), which can respond to the reduction of CO_2_ assimilation capacity by actively regulating the electron transfer efficiency and photochemical efficiency and avoiding or mitigating the damage to the photosynthesis system caused by excess light energy in the form of heat dissipation and other forms of energy, which allows chlorophyll fluorescence technology to probe the more in‐depth rainfall changes on plant photosynthesis (Bu, Zhang, and Chang 2010; Yang, Lang, and Zhang 2018). A study on the effect of moisture changes on the fluorescence parameters of *Alchornea trewioides* in the karst region found that leaf fluorescence parameter F0 increased significantly with decreasing precipitation, and Fm, Fv/F0, and Fv/Fm decreased gradually (Zhao, Wang, et al. 2023; Zhao, Wu, et al. 2023). It has also been shown that the maximum photochemical quantum yield (Fv/Fm) of PSII of *Platycladus orientalis* seedlings showed a decreasing trend with decreasing precipitation (Zhang et al. 2021). Meanwhile, the decrease in precipitation reduced the efficiency and activity of PSII primary light energy conversion and photosynthetic electron transfer in *Forsythia suspensa* (Lang et al. 2018). Accordingly, the study of changes in fluorescence parameters in plants under changing rainfall patterns will be useful in understanding the changes in their photosynthetic apparatus, which is important for their accumulation of substances under different environmental conditions.

*Fraxinus malacophylla* is a plant belonging to the genus *Fraxinus* of Oleaceae, which is light‐loving, cold‐ and drought‐resistant, and barren‐resistant. It is mainly distributed in Yunnan and Guangxi, China, and is one of the preferred tree species to promote ecological restoration in rocky desertification areas (Zhang, Dong, et al. 2022; Zhang, Li, et al. 2022; Zheng, Duan, and Dong 2023). *Fraxinus malacophylla* has strong adaptability and a high afforestation survival rate in rocky desertified mountains, and it not only has important ecological value but also has high medicinal, economic, and ornamental values (Guo et al. 2023). It is often used to treat constipation, malaria, epilepsy, and other diseases (Duan et al. 2020); its wood can be used as furniture, agricultural tools, and handles of artifacts (Jing et al. 2022); in addition, with its beautiful shape and luxuriant foliage, it can be used as an ornamental tree species for greening (Zhang 2010). As one of the major native tree species in Southwest China, *Fraxinus malacophylla* plays a pivotal role in the ecological recovery of the rocky desertification area in Southwest China (Duan et al. 2022). Currently, research on *Fraxinus malacophylla* mainly focuses on silvicultural techniques (Li, Dong, et al. 2017; Li, Tang, et al. 2017), soil moisture, and nutrients (Xia et al. 2016; Huan et al. 2019). Though the dry and rainy seasons are visible in southwest China, it is still unknown how the leaf features and photosynthetic fluorescence characteristics of *Fraxinus malacophylla* seedlings react to rainfall patterns in that area. Consequently, this study is of great significance to improve the water management efficiency of *Fraxinus malacophylla* in Southwest China and to accelerate the promotion of rocky desertification recovery in Southwest China. This study aims to answer the following questions: (1) How do rainfall increases and decreases during the dry and rainy seasons affect leaf traits, photosynthetic parameters, and fluorescence parameters of *Fraxinus malacophylla* seedlings; and is there a phenomenon that is facilitated by rainfall increases and inhibited by rainfall decreases? (2) How do the leaf traits, photosynthetic parameters, and fluorescence parameters of *Fraxinus malacophylla* seedlings adapt to the interval of rainfall, and is prolonged rainfall facilitated or inhibited? (3) Associations among leaf traits, photosynthetic parameters, and fluorescence parameters of *Fraxinus malacophylla* seedlings under rainfall patterns.

## 2 Materials and Methods

### 2.1 Overview of the Study Area

**FIGURE 1:**
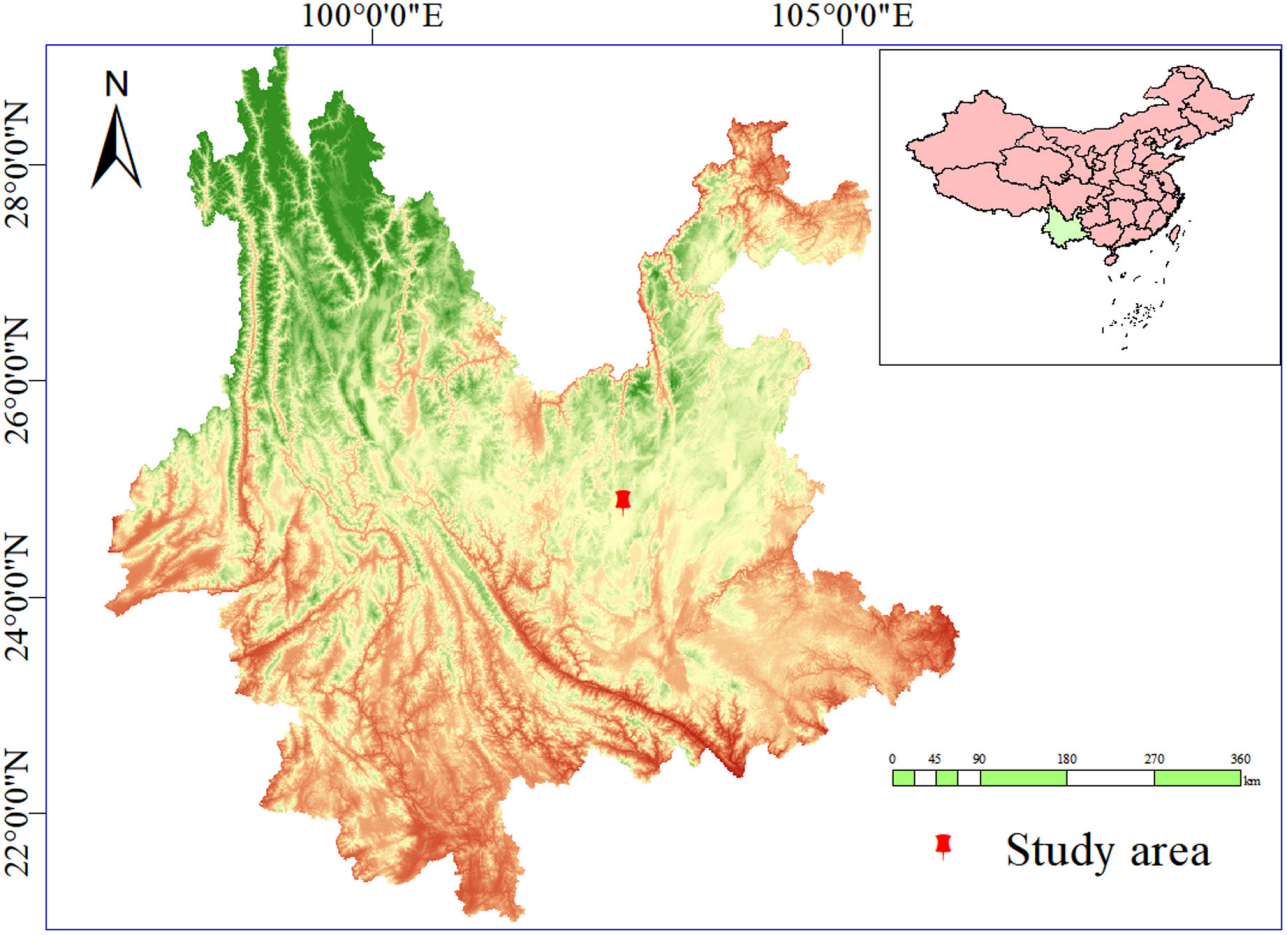
Geo‐referenced map of the study area in Southwest China.

The study was carried out in a greenhouse at the Southwest Forestry University of China (Kunming, Yunnan, 102° 46′ E, 25° 03′ N; Figure 1). Located in the Yunnan‐Guizhou Plateau, it has a climate that is classified as a northern subtropical low‐latitude semi‐moist plateau mountain monsoon. The area is mountainous, with red loam soil. The average annual temperature is 16.5°C, the average annual precipitation is 700–1100 mm, with rainfall concentrated in May through October, and the average annual relative humidity is 67%.

### 2.2 Test Materials

#### 2.2.1 Fraxinus malacophylla

**FIGURE 2:**
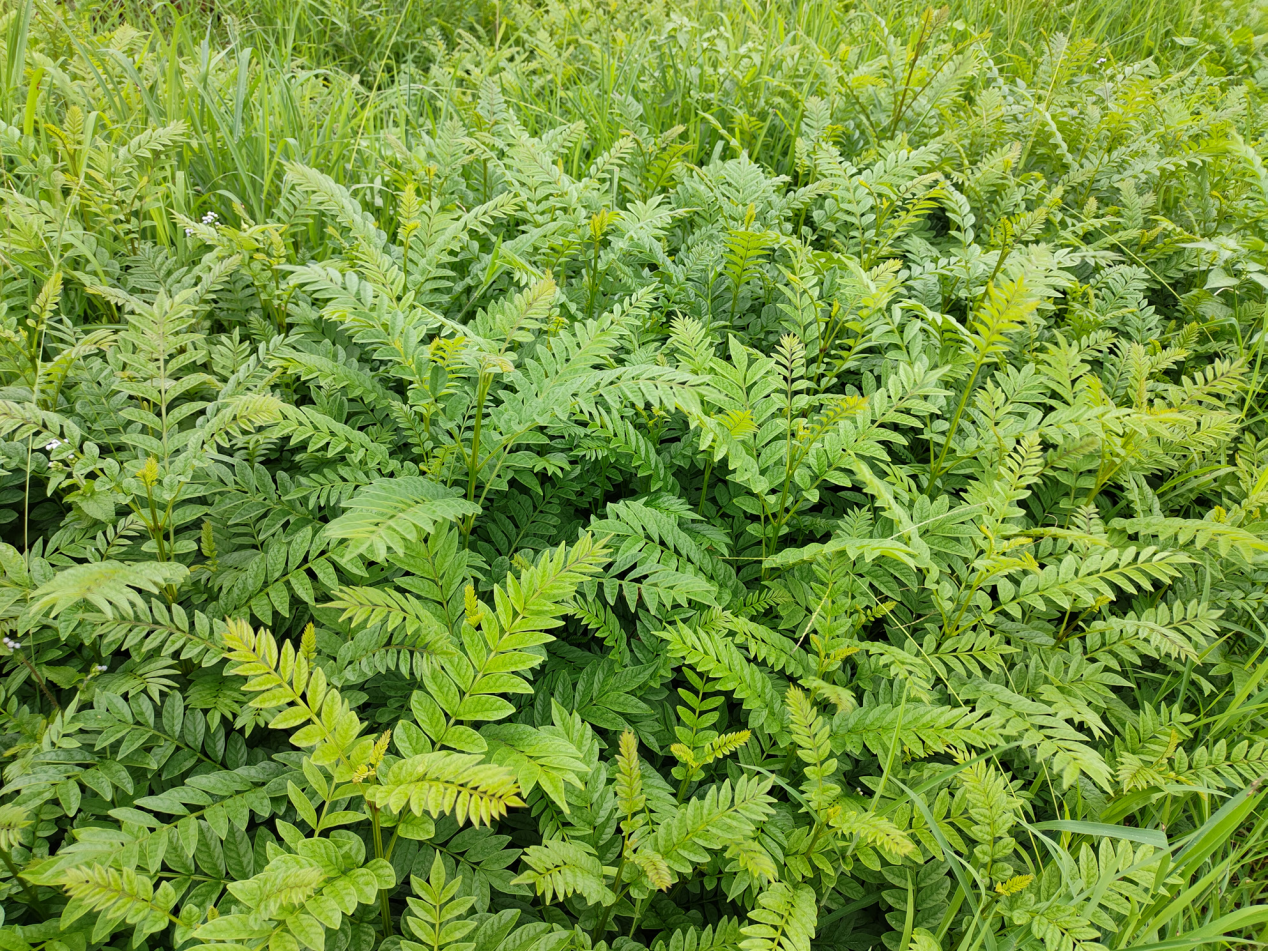
*Fraxinus malacophylla*. Branchlets are sparsely pilose and velutinous, pinnately compound, and leaflets are thinly leathery. Landscaping species and rocky desertification control species.

Branchlets are sparsely pilose and velutinous, pinnately compound, and leaflets are thinly leathery. Landscaping species and rocky desertification control species (Figure 2). The plant material used in this experiment was 2‐year‐old seedlings of *Fraxinus malacophylla* provided by Yunnan Jianshui Chengfa Greening Co. On June 8, 2022, the seedlings were obtained from Southwest Forestry University. Following 90 days of seedling refining, the seedlings were moved into containers measuring 20 cm in the upper caliber, 14 cm in the lower caliber, and 18 cm in height. They were then acclimated to growing in a greenhouse shed located in Green. The test soil consisted of a mixture of red loam, humus, and perlite in a ratio of 5:3:2. The potting soil had a field water holding capacity of 26.43%, a pH of 5.52, a bulk density of 1.31 g/cm^3^, soil organic matter of 3.23 g/kg, organic carbon of 32.43 g/kg, total nitrogen of 0.86 g/kg, total phosphorus of 0.41 g/kg, hydrolyzed nitrogen of 45.62 mg/kg, and quick‐acting phosphorus of 11.78 mg/kg. Each pot was filled with one well‐grown seedling.

### 2.3 Experimental Design

In this study, a two‐factor randomized area group test was employed. The first factor is the rainfall interval, which is represented by the letters T and T_+_, respectively, as 5 days for the natural rainfall interval and 10 days for the extended rainfall interval. Factor 2 is rainfall. Based on meteorological data from Duan, Wang, and Wang (2019) and Li and He (2016), rainfall spatial and temporal distribution research reveals that: Yunnan Kunming's average annual rainfall from 1988 to 2017 was 975.5 mm; the most precipitation‐producing year was 1999 with 1449.9 mm, 45% more than previous years; the least precipitation‐producing year was 2009 with 565.8 mm, 42% less than previous years; the maximum annual precipitation is 1.30–1.60 times the multi‐year average precipitation; and the minimum annual precipitation is 0.47–0.71 times the multi‐year average precipitation. As a result, the average monthly rainfall (W) served as the control, and the corresponding rainfall treatments were 40% higher (W_+_) and 40% lower (W_−_). Table 1 shows the exact amounts of rainfall. There were six treatments in the experiment, each with three zones and 16 seedlings per zone, for a total of 288 plants. The following treatments are available: 1. W‐T, 2. WT, 3. W_+_T, 4. W‐T_+_, 5. WT_+_, and 6. W_+_T_+_. Water control experiments were carried out in the greenhouse after 50 days of acclimation, from October 28, 2022, to September 28, 2023.

**TABLE 1:** Water control test design table.

Month	Monthly average rainfall/mm	Watering intervals/d	Monthly watering frequency	Single watering amount/mL		
				W_−_	W	W_+_
November to January (At the beginning of the dry season)	25.41	T T_+_	6 3	80 160	133 266	186 372
February to April (At the end of the dry season)	20.34	T T_+_	6 3	63 127	106 212	148 297
May to July (At the beginning of the rainy season)	146.76	T T_+_	6 3	460 921	768 1536	1075 2150
August to October (At the end of the rainy season)	150.06	T T_+_	6 3	471 942	785 1570	1099 2199

*Note:* T, rainfall interval time is 5 days; T_+_, rainfall interval time is 10 days; W, average monthly rainfall; W_−_, water reduction 40%; W_+_, water addition 40%.

### 2.4 Sample Collection and Data Measurement

#### 2.4.1 Measurement of Trait Indicators

Three seedlings were randomly selected on December 28, March 28, June 28, and September 28 for each treatment. The number of leaves of each plant was counted, and the leaf length, leaf width, leaf perimeter, and leaf area of each leaf of a single plant were determined by using the YMJ‐C Intelligent Leaf Area Measuring Instrument (TOP, China), and the leaf area of a single plant was calculated. Then wash with water and filter paper to absorb the moisture, cut and mix the leaves of *Fraxinus malacophylla* seedlings into envelopes and mark them well to bring them back to the laboratory to obtain the fresh weight of the leaves, put them in the oven at a temperature of 105°C for 30 min, and then adjust to 80°C to dry to a constant weight, and then weigh them to get the dry weight of the leaves. The leaf shape index, leaf water content, specific leaf area (SLA), and specific leaf mass (LMA) were calculated according to the following formulas:

(1) Leaf shape index=leaf length/leaf width

(2) $$\text{Leaf water content}=\left(\text{fresh weight}-\mathrm{dry}\;\text{weight}\right)/\text{fresh weight}$$

(3) $$\mathrm{SLA}=\text{leaf area}\;\left({\mathrm{cm}}^{2}\right)/\text{leaf}\;\mathrm{dry}\;\text{weight}\;\left(g\right)$$

(4) $$\mathrm{LMA}=\text{Leaf blade}\;\mathrm{dry}\;\text{weight}\;\left(g\right)/\text{Leaf blade area}\;\left({\mathrm{cm}}^{2}\right)$$

#### 2.4.2 Measurement of Photosynthetic Parameters

The photosynthesis parameters of *Fraxinus malacophylla* were measured by the LI‐6800 portable photosynthesizer (LI‐COR, USA), and the daily changes in photosynthesis of the 4th–6th complete functional leaves counted down from the terminal bud were measured from 8:00 to 18:00, and the results were measured once at an interval of 2 h. Three seedlings of the same growth condition were selected for each treatment, and the results were averaged out. For each treatment, three seedlings with almost the same growth condition were selected, 22 leaves were measured for each plant, each leaf was repeated three times (10 values were read each time), and the results were averaged. During the same period, the photosynthetic characteristics were measured at 9:30–11:30 am on a sunny day, and the indicators included photosynthetic rate (Pn), stomatal conductance (Gs), intercellular CO_2_ concentration (Ci), and transpiration rate (Tr) of the functional leaves of *Fraxinus malacophylla* seedlings. The water use efficiency (WUE) was calculated:

(5) WUE = Pn/Tr

#### 2.4.3 Measurement of Fluorescence Parameters

Three seedlings of uniform growth were selected for each treatment, and two leaves of the 4th to 6th leaves that were free of pests and diseases, well‐grown, and functionally intact were selected from each plant for labeling. Chlorophyll fluorescence parameters were determined using a PEA‐Plus high‐speed continuous excitation fluorometer (Hansatech, UK), which was dark‐treated for 30 min, and initial fluorescence yield (F0), maximum fluorescence yield (Fm), maximum quantum yield of PSII (Fv/Fm), and PSII performance indices based on light absorption (PIabs) were determined three times per leaf for 3 days and averaged.

#### 2.4.4 Data Processing

Data were processed using Excel 2016; geo‐referenced mapping of the study area using ArcMap 10.6 (ESRI., Redlands, CA, USA); two‐way ANOVA was performed using SPSS 27.0 (SPSS Inc., Chicago, IL, USA) to compare the effects of changes in rainfall patterns on leaf trait indexes, photosynthetic parameters, and fluorescence parameters of *Fraxinus malacophylla* seedlings; multiple comparisons and significance analyses were performed using the LSD test; and graphs were prepared using Origin 2021 (OriginLabCo., Northampton, MA, USA).

## 3 Results

### 3.1 Leaf Trait Characteristics

As shown in Table 2, there were both significant and non‐significant differences between the rainfall patterns (rainfall interval and rainfall amount) and the interaction of rainfall interval and rainfall amount for each of the leaf traits of *Fraxinus malacophylla* seedlings during the dry and rainy season periods. The non‐significant differences were predominant, and the significant differences were low. Under the 10‐day treatment at the beginning of the dry season (December), the rain‐reduced plants showed smaller leaf length and lower SLA, and the rain‐increased plants showed larger leaf length and higher SLA. In the dry season, when the rainfall interval was unchanged, the leaf blades more often showed larger leaf area, leaf shape index, etc. under the natural rainfall. Under the natural rainfall at the beginning of the rainy season (June), the leaf water content at 10 days was significantly higher than that at 5 days (*p* < 0.05), elevated by 63.64%, and the rain increment in the rainy season made the leaves show higher water content, and at the same time, the rain‐boosted plants had a larger leaf perimeter and leaf area.

**TABLE 2:** Characteristics of leaf trait changes.

Norm	Address		Months			
			December	March	June	September
Number of leaves (pieces)	5 days	W_−_	18.00 ± 5.29a	14.00 ± 3.46ab	11.67 ± 1.53a	9.00 ± 1.00 cd
		W	17.67 ± 4.93a	13.33 ± 4.51ab	6.33 ± 0.58b	14.00 ± 3.46a
		W_+_	16.33 ± 6.66a	19.33 ± 4.51a	8.67 ± 2.08ab	11.67 ± 1.15abc
	10 days	W_−_	14.67 ± 2.31a	10.33 ± 2.08b	9.33 ± 2.31ab	12.67 ± 2.08ab
		W	13.00 ± 2.65a	13.67 ± 6.66ab	9.33 ± 1.53ab	7.33 ± 0.58d
		W_+_	16.33 ± 2.52a	13.00 ± 3.00ab	11.00 ± 1.00a	10.33 ± 0.58bcd
	W		0.902	0.295	0.036*	0.950
	T		0.221	0.137	0.214	0.114
	W × T		0.647	0.425	0.029*	0.001**
Leaf length (cm)	5 days	W_−_	9.72 ± 1.47a	13.49 ± 2.44ab	15.27 ± 4.43a	16.68 ± 1.49a
		W	11.78 ± 4.10a	14.19 ± 1.41a	17.14 ± 2.45a	16.42 ± 1.61ab
		W_+_	11.77 ± 2.75a	10.60 ± 1.40b	16.21 ± 1.59a	15.34 ± 0.78ab
	10 days	W_−_	12.48 ± 1.75a	14.18 ± 1.61a	16.28 ± 2.37a	12.20 ± 4.76b
		W	14.26 ± 1.38a	13.42 ± 1.39ab	16.47 ± 2.10a	16.99 ± 0.09a
		W_+_	11.74 ± 1.95a	10.28 ± 2.26b	15.26 ± 2.87a	16.09 ± 1.71ab
	W		0.405	0.010*	0.758	0.265
	T		0.154	0.877	0.879	0.345
	W × T		0.563	0.776	0.807	0.118
Leaf width (cm)	5 days	W_−_	4.43 ± 2.03a	8.61 ± 1.29a	6.99 ± 2.49a	6.50 ± 2.32a
		W	4.97 ± 2.07a	7.19 ± 1.19ab	8.61 ± 1.30a	6.94 ± 0.64a
		W_+_	5.35 ± 2.05a	5.52 ± 1.36b	7.22 ± 0.37a	7.21 ± 1.63a
	10 days	W_−_	5.62 ± 0.93a	8.06 ± 1.11a	8.65 ± 1.41a	6.30 ± 0.91a
		W	7.09 ± 1.24a	7.19 ± 0.32ab	8.77 ± 1.28a	7.57 ± 1.48a
		W_+_	4.40 ± 0.12a	6.96 ± 1.35ab	6.34 ± 1.28a	8.01 ± 1.66a
	W		0.414	0.028*	0.125	0.406
	T		0.312	0.595	0.664	0.582
	W × T		0.265	0.341	0.364	0.837
Leaf shape index	5 days	W_−_	2.38 ± 0.64a	1.57 ± 0.16a	2.24 ± 0.25ab	2.78 ± 0.89a
		W	2.44 ± 0.53a	2.03 ± 0.49a	2.01 ± 0.31ab	2.37 ± 0.04a
		W_+_	2.32 ± 0.48a	1.96 ± 0.27a	2.26 ± 0.33ab	2.20 ± 0.45a
	10 days	W_−_	2.29 ± 0.67a	1.80 ± 0.46a	1.89 ± 0.06b	1.89 ± 0.54a
		W	2.03 ± 0.18a	1.87 ± 0.19a	1.88 ± 0.06b	2.31 ± 0.50a
		W_+_	2.66 ± 0.37a	1.51 ± 0.43a	2.42 ± 0.19a	2.04 ± 0.23a
	W		0.682	0.428	0.029*	0.706
	T		0.824	0.472	0.343	0.156
	W × T		0.466	0.288	0.188	0.343
Leaf perimeter (cm)	5 days	W_−_	51.30 ± 10.41c	75.51 ± 12.40b	93.63 ± 10.27a	131.90 ± 10.76a
		W	57.71 ± 15.72bc	69.58 ± 12.11b	121.83 ± 15.59a	90.05 ± 7.15b
		W_+_	74.88 ± 7.02b	53.38 ± 9.13b	120.65 ± 75.88a	96.51 ± 27.24ab
	10 days	W_−_	69.43 ± 9.54bc	122.42 ± 14.60a	128.81 ± 32.82a	82.15 ± 31.19b
		W	128.27 ± 15.06a	85.07 ± 21.28b	130.01 ± 31.12a	120.52 ± 20.93ab
		W_+_	57.32 ± 9.15bc	65.24 ± 25.24b	90.38 ± 16.00a	117.96 ± 12.66ab
	W		0.001**	0.005**	0.633	0.983
	T		0.001**	0.009**	0.809	0.941
	W × T		0.000***	0.180	0.347	0.010*
Leaf area (cm^2^)	5 days	W_−_	521.02 ± 58.11b	514.94 ± 105.67bc	1038.44 ± 196.51a	626.53 ± 80.41a
		W	590.33 ± 137.79ab	809.87 ± 84.62a	400.28 ± 76.48b	723.36 ± 69.91a
		W_+_	481.25 ± 84.78b	585.71 ± 27.32b	517.77 ± 176.09b	599.22 ± 146.97ab
	10 days	W_−_	540.13 ± 92.77b	818.61 ± 21.53a	476.87 ± 127.42b	451.36 ± 65.64b
		W	756.98 ± 106.13a	562.86 ± 51.71bc	641.65 ± 173.75b	441.04 ± 89.19b
		W_+_	523.02 ± 114.34b	441.48 ± 77.13c	517.63 ± 111.82b	598.46 ± 47.83ab
	W		0.028*	0.002**	0.025*	0.504
	T		0.141	0.383	0.156	0.003**
	W × T		0.429	0.000***	0.002**	0.052
Leaf water content	5 days	W_−_	0.55 ± 0.03ab	0.52 ± 0.09a	0.41 ± 0.12ab	0.52 ± 0.01a
		W	0.41 ± 0.09c	0.43 ± 0.18a	0.33 ± 0.08b	0.45 ± 0.15a
		W_+_	0.59 ± 0.04ab	0.52 ± 0.06a	0.42 ± 0.04ab	0.57 ± 0.08a
	10 days	W_−_	0.40 ± 0.04c	0.36 ± 0.12a	0.38 ± 0.07b	0.52 ± 0.04a
		W	0.66 ± 0.06a	0.33 ± 0.21a	0.54 ± 0.07a	0.55 ± 0.04a
		W_+_	0.53 ± 0.09b	0.43 ± 0.09a	0.40 ± 0.05ab	0.57 ± 0.02a
	W		0.117	0.484	0.620	0.234
	T		0.639	0.097	0.153	0.349
	W × T		0.000***	0.876	0.027*	0.458
SLA (cm^2^ g^−1^)	5 days	W_−_	94.53 ± 11.58ab	116.34 ± 27.18bc	139.51 ± 26.71a	66.59 ± 8.97ab
		W	84.48 ± 25.94b	211.13 ± 81.93a	42.79 ± 6.58c	72.28 ± 9.60a
		W_+_	78.50 ± 20.28b	126.52 ± 40.47bc	51.56 ± 14.75bc	53.12 ± 17.77ab
	10 days	W_−_	97.53 ± 22.89ab	180.39 ± 47.87ab	69.73 ± 22.11bc	49.80 ± 12.62ab
		W	131.59 ± 23.18a	98.42 ± 11.89bc	89.09 ± 30.59b	48.29 ± 17.03ab
		W_+_	94.67 ± 23.79ab	90.23 ± 7.68c	63.24 ± 6.06bc	46.23 ± 6.79b
	W		0.270	0.183	0.004**	0.348
	T		0.052	0.197	0.686	0.022*
	W × T		0.238	0.015*	0.001**	0.528
LMA (g cm^−2^)	5 days	W_−_	0.011 ± 0.001a	0.009 ± 0.002ab	0.007 ± 0.002d	0.015 ± 0.002a
		W	0.013 ± 0.004a	0.005 ± 0.002b	0.024 ± 0.004a	0.014 ± 0.002a
		W_+_	0.013 ± 0.004a	0.009 ± 0.003ab	0.020 ± 0.005ab	0.021 ± 0.008a
	10 days	W_−_	0.011 ± 0.003a	0.006 ± 0.002b	0.016 ± 0.006bc	0.021 ± 0.005a
		W	0.008 ± 0.001a	0.010 ± 0.001a	0.012 ± 0.005 cd	0.023 ± 0.010a
		W_+_	0.011 ± 0.003a	0.011 ± 0.001a	0.016 ± 0.002bc	0.022 ± 0.003a
	W		0.472	0.097	0.024*	0.623
	T		0.105	0.120	0.208	0.075
	W × T		0.377	0.009**	0.004**	0.545

*Note:* The months of December and March are the dry season period, June and September are the rainy season period. All data in the table are mean ± standard deviation. Different lowercase letters in the same column are significant differences between different treatment groups (*p* < 0.05). W: rainfall amount, T: rainfall interval, W × T: rainfall amount × rainfall interval. The corresponding positions in the table represent two‐factor ANOVA.

*p < 0.05, **p < 0.01, ***p < 0.001.

Overall, leaf length, leaf width, leaf perimeter, and LMA were higher in the rainy season than in the dry season and were mostly optimal under natural rainfall, and the extended rainfall interval was higher than the natural rainfall interval. The reason for this is that increased rainfall and multiple rainfalls tend to lead to flooding of *Fraxinus malacophylla*, insufficient supply of nutrients, slow growth, and growth inhibition, resulting in slow leaf growth during natural rainfall hours, whereas reduced rainfalls will make the leaves dehydrated, photosynthesis weakened, and less organic matter made, which will likewise impede the growth of the leaves. This also indicates that *Fraxinus malacophylla* seedlings can adapt to some drought. At the same time, the leaf number and SLA in the dry season were higher than those in the rainy season, which also indicated that plants adapted to drought by increasing transpiration through high leaf numbers in the dry season.

### 3.2 Leaf Photosynthesis Characteristics

**FIGURE 3:**
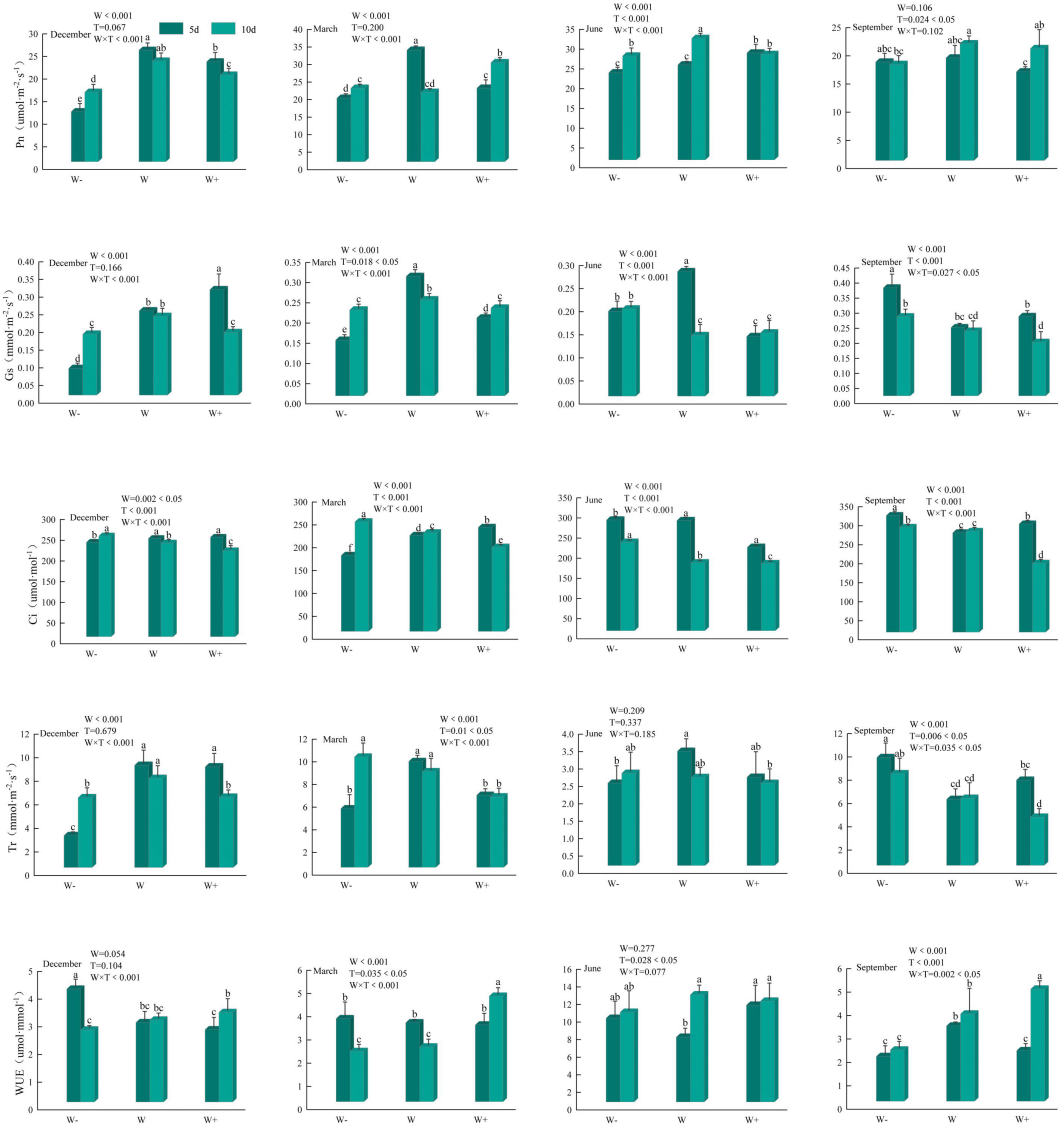
Characterization of changes in leaf photosynthetic parameters. The months of December and March are the dry season period, June and September are the rainy season period. Different lowercase letters indicate significant differences between treatments (*p* < 0.05). W: Rainfall amount, T: Rainfall interval, W × T: Rainfall amount × rainfall interval. The corresponding positions in the figure represent two‐factor ANOVA.

The photosynthetic parameters of the leaves of *Fraxinus malacophylla* seedlings differed under different rainfall patterns in the dry and rainy seasons (Figure 3). During the dry season, all leaf photosynthetic parameters were significantly different (*p* < 0.01) under the interaction of rainfall interval and rainfall amount, and all photosynthetic parameters except WUE at the beginning of the dry season were significantly different (*p* < 0.01) under the rainfall amount treatment, whereas both *p* < 0.05 and *p* > 0.05 existed under the rainfall interval treatment; during the rainy season, all photosynthetic parameters were significantly different (*p* < 0.05) under the rainfall pattern (rainfall interval versus rainfall amount) and rainfall amount treatment, except for Pn at the end of the rainy season (September), WUE at the beginning of the rainy season, Tr, and WUE. Tr, and WUE, there was significant (*p* < 0.05) variability in all photosynthetic parameters of leaves under rainfall pattern (rainfall interval vs. rainfall amount) and rainfall interval vs. rainfall amount interaction.

The photosynthetic parameters of the leaves of *Fraxinus malacophylla* seedlings were characterized by different changes under different rainfall patterns (Figure 3). In the dry season, Pn, Gs, and Tr of plant leaves basically showed an increasing and then decreasing trend with the increase of rainfall and generally reached the highest level under natural rainfall, indicating that there is a suitable amount of water demand for leaves, and exceeding or falling below this amount would weaken photosynthesis and inhibit plant growth; however, with the increase of rainfall, natural rainfall versus the decrease of rainfall, and the length of natural rainfall versus the prolonged rainfall, most of the leaf photosynthetic parameters remained at a high level. Most of the leaf photosynthetic parameters remained high; for WUE, it showed a decreasing trend with the increase of rainfall in 5 days of treatment and an increasing trend with the increase of rainfall in 10 days of treatment; for Ci, it showed an increasing trend with the increase of rainfall in 5 days of treatment and a decreasing trend with the increase of rainfall in 10 days of treatment, that is, there is a phenomenon that Ci increases when Pn decreases. The Pn of plant leaves under 5 days treatment at the beginning of the rainy season tended to increase with the increase of rainfall, and the Ci under 5 days treatment was significantly higher than that under 10 days (*p* < 0.05) and elevated by 25.12%, 62.19%, and 24.37% under reduced, natural, and increased rainfall, respectively; for Gs, Ci, and Tr during the rainy season period, basically, they all showed a decreasing trend with the increase of rainfall, and reached the highest, and the natural rainfall interval was higher than the prolonged rainfall interval, indicating that in the rainy season plant leaves demand less water and demand water more often, that is, reduced rainfall and multiple rainfalls tend to promote plant photosynthesis; for WUE, the WUE of 10 days was higher than that of 5 days, suggesting that the decrease in the frequency of rainfall increases the demand for water utilization by *Fraxinus malacophylla*.

### 3.3 Leaf Fluorescence Characteristics

**FIGURE 4:**
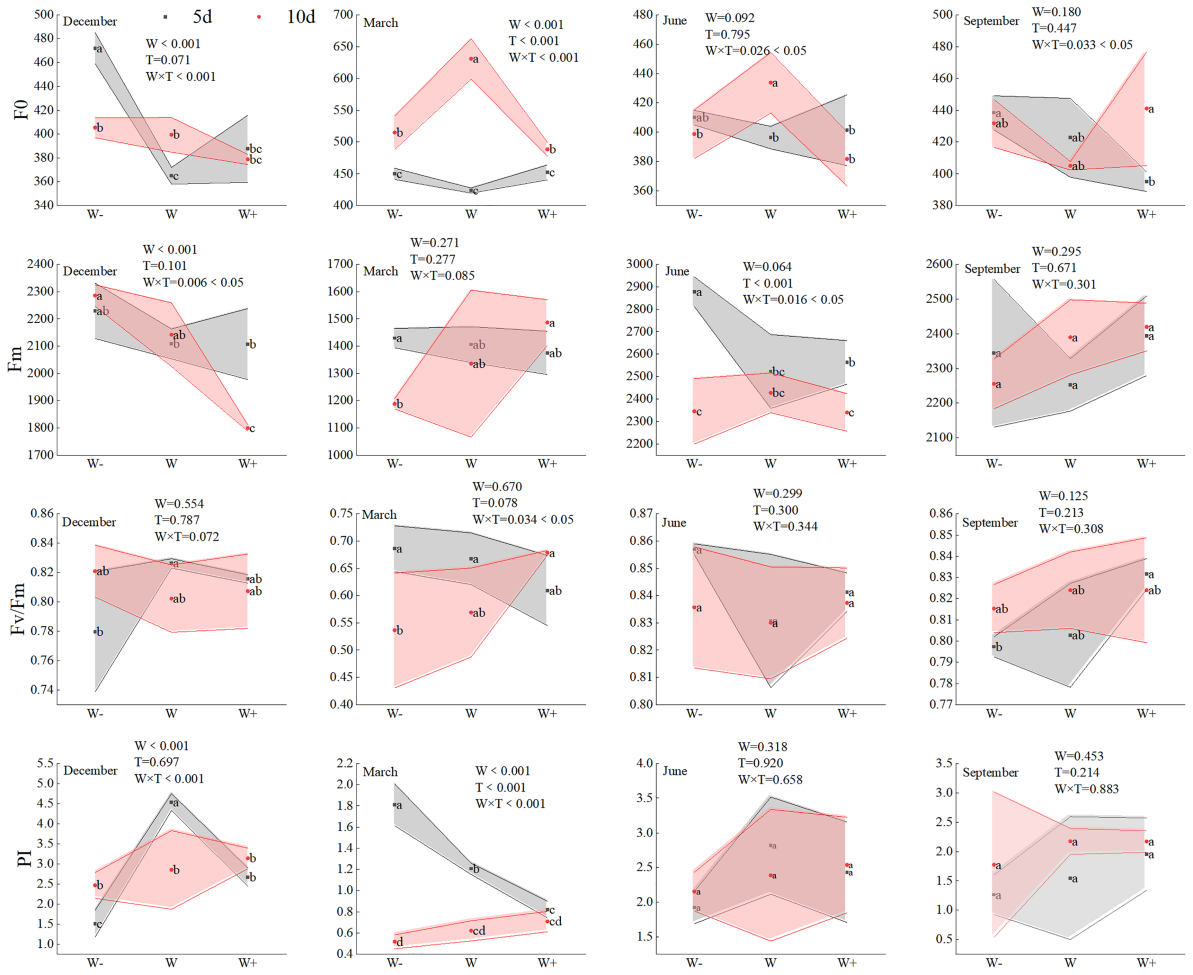
Characterization of changes in leaf fluorescence parameters. The months of December and March are the dry season period, June and September are the rainy season period. Different lowercase letters indicate significant differences between treatments (*p* < 0.05). W: Rainfall amount, T: Rainfall interval, W × T: Rainfall amount × rainfall interval. The corresponding positions in the figure represent two‐factor ANOVA.

There were some significant differences in the fluorescence parameters of *Fraxinus malacophylla* seedling leaves under different rainfall patterns in the dry and rainy seasons (Figure 4). During the dry season, all the fluorescence parameters of leaves were significantly different (*p* < 0.05) under the interaction of rainfall interval and rainfall amount except for Fv/Fm at the beginning of the dry season and Fm at the end of the dry season, rainfall interval was significantly different (*p* < 0.01) only for F0 and PI at the end of the dry season, and for rainfall amount, all of them were also significantly different (*p* < 0.01) except for Fv/Fm as well as Fm at the end of the dry season; for the rainy season period, all fluorescence parameters were not significantly different (*p* > 0.05) under each treatment except for F0 and Fm at the beginning of the rainy season, which were significantly different (*p* < 0.05) under the interaction of rainfall interval and rainfall amount, and Fm at the beginning of the rainy season, which was significantly different (*p* < 0.01) under the rainfall interval treatment.

The fluorescence parameters of *Fraxinus malacophylla* seedling leaves were characterized by different changes under different rainfall patterns (Figure 4). At the beginning of the dry season, F0 under 5 days treatment showed a significant trend of decreasing and then increasing with the increase of rainfall (*p* < 0.05), and it was 29.32% and 21.75% higher than that of natural rainfall and rain increment, respectively; PI under 5 days treatment showed a trend of increasing and then decreasing with the increase of rainfall, with a significant difference (*p* < 0.05), and it reached a peak under natural rainfall, which was 3.00 times that of rain increment and 70.11% higher than that of rain decrement. At the end of the dry season, the Fv/Fm at 5 days was significantly higher than that at 10 days under the reduced rainfall treatment (*p* < 0.05), elevated by 27.91%, and the leaf Fv/Fm was lower than 0.80 under all treatment groups; for PI, the PI at 5 days was higher than that at 10 days, and the PI value was higher under the natural precipitation in the whole dry season. During the rainy season period, Fv/Fm was not significantly different (*p* > 0.05) under each treatment group, and the values of Fv/Fm tended to range from 0.80 to 0.83. At the beginning of the rainy season, Fm was lower at 10 days than at 5 days. At the end of the rainy season, all fluorescence parameters of *Fraxinus malacophylla* leaves were not significantly different (*p* > 0.05) under each treatment group.

### 3.4 Correlation Analysis

**FIGURE 5:**
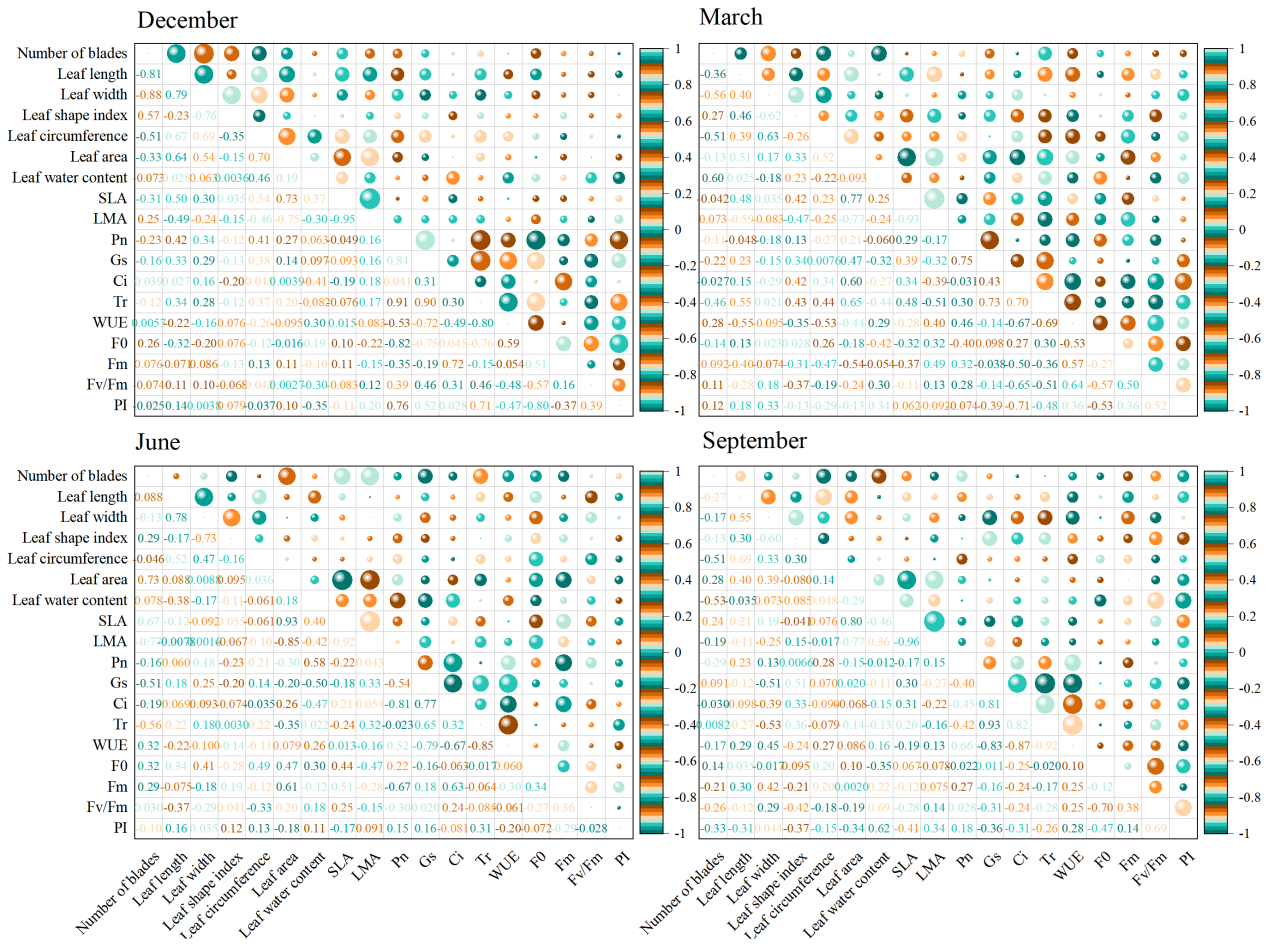
Heat map of correlation between leaf traits, photosynthetic parameters and fluorescence parameters. The months of December and March are the dry season period; June and September are the rainy season period. Larger and darker spheres represent a stronger positive or negative correlation between the two variables.

Correlations between leaf traits, photosynthetic parameters, and fluorescence parameters were found when analyzing the different rainfall patterns of *Fraxinus malacophylla* seedlings during the dry and rainy seasons (Figure 5). At the beginning of the dry season, leaf length and leaf width of *Fraxinus malacophylla* seedlings were positively correlated; Pn was closely and positively correlated with Gs and Tr; Gs was closely and positively correlated with Tr; Ci was positively correlated with Fm; Pn and Tr were positively correlated with PI; and Pn, Gs, and Tr were negatively correlated with F0. At the end of the dry season, leaf width was positively correlated with leaf perimeter; Pn was closely and positively correlated with Gs, and Gs and Ci were closely and positively correlated with Tr; Ci and Tr were negatively correlated with WUE. At the beginning of the rainy season, leaf width was closely and positively correlated with leaf length; Gs was closely and positively correlated with Ci and Tr; Pn was closely and negatively correlated with Ci; and Gs, Ci, and Tr were all negatively correlated with WUE. At the end of the rainy season, leaf length was closely and positively correlated with leaf perimeter; Gs was closely and positively correlated with Ci and Tr; Ci was closely and positively correlated with Tr; Pn was positively correlated with WUE; and Gs, Ci, and Tr were all closely and negatively correlated with WUE. In conclusion, there was a close association between photosynthetic and fluorescence parameters of *Fraxinus malacophylla* seedlings, while the relationship between leaf traits and photosynthetic fluorescence parameters was not significant, which may be attributed to the narrow leaves of *Fraxinus malacophylla* seedlings in the face of seasonal and rainfall variations, as well as in an environment of water deficit or excess water, which affects the linkage with the photosynthetic system, whereas the phytofluorescence system directly affects photosynthesis.

### 3.5 Principal Components Analysis

**FIGURE 6:**
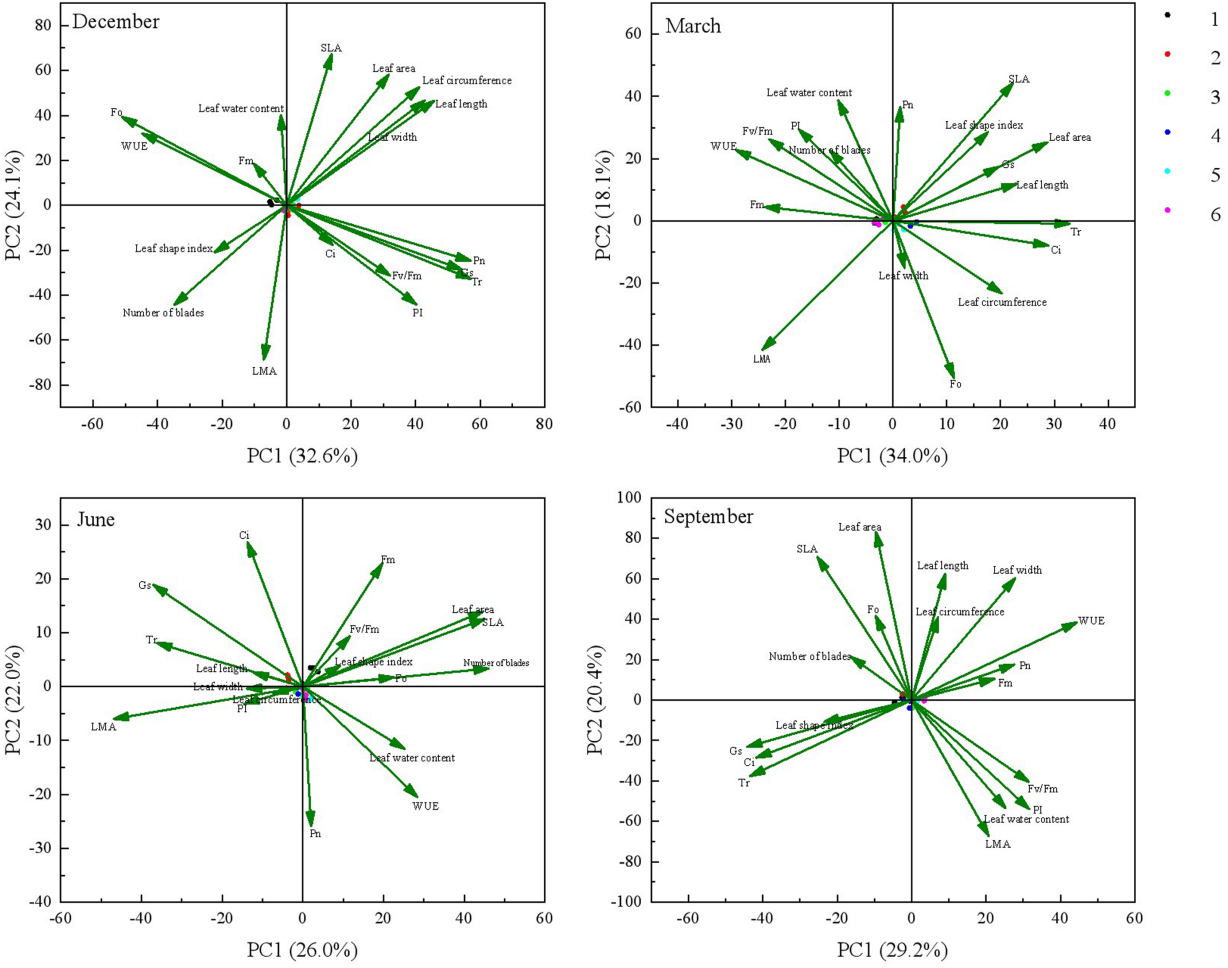
Principal component analysis (PCA) biplots for leaf traits, photosynthetic parameters, and fluorescence parameters. The months of December and March are the dry season period; June and September are the rainy season period. Vertical and horizontal coordinates represent the correlation between variables and principal components 1 (PC1) and 2 (PC2), respectively. The distances between the variables show the strength of their correlation; the distances between the variables and the horizontal axis, and between the variables and the vertical axis show the relationship between them, respectively. 1. W_−_T, 2. WT, 3. W_+_T, 4. W_−_T_+_, 5. WT_+_, and 6. W_+_T_+_.

As shown in Figure 6, the principal component relationships of leaf traits, photosynthetic parameters, and fluorescence parameters of *Fraxinus malacophylla* seedlings behaved differently under different rainfall pattern treatments in the dry and rainy seasons. At the beginning of the dry season, the PC1 axis was mainly related to Pn, Gs, Tr, WUE, and F0, and the PC2 axis was mainly related to leaf water content, SLA, and LMA. At the end of the dry season, the PC1 axis was mainly related to leaf length, leaf area, Gs, Tr, WUE, Fm, Fv/Fm, and PI, and the PC2 axis was mainly related to leaf water content, Pn, and F0. At the beginning of the rainy season, the PC1 axis is mainly related to the number of leaves, leaf area, SLA, LMA, and Tr, while the PC2 axis is mainly related to Pn, Ci, and Fm. At the end of the rainy season, the PC1 axis is mainly related to Gs, Ci, and Tr, and the PC2 axis is mainly related to leaf length, leaf area, and LMA. In summary, leaf water content, Pn, Gs, Tr, WUE, and F0 were the main components of *Fraxinus malacophylla* under different rainfall pattern treatments in the dry season, which represented most of the information, this suggests that water‐related indicators dominate in the dry season, probably because *Fraxinus malacophylla* are under‐supplied with water in the dry season, and are therefore sensitive to changes in water‐closely related physiological activities; while leaf area, LMA, Ci, and Tr were the main components under different rainfall pattern treatments in the rainy season, which represented most of the information, this is because of the abundant precipitation during the rainy season, the plant absorbs CO_2_, which promotes photosynthesis and the accumulation of organic matter, prompting the growth of leaves.

## 4 Discussion

### 4.1 Leaf Trait Characteristics

As one of the important organs of plants for photosynthesis, respiration, transpiration, etc., leaf traits can well respond to the adaptive responses of plants to the external environment (Akram et al. 2023). And water influences biochemical reactions and metabolism, regulates plant growth, affects productivity, and influences ecosystems (Anderson‐Teixeira et al. 2011; Sibly, Brown, and Kodric‐Brown 2012; Huang, Ran, Ji, et al. 2020; Huang, Ran, Li, et al. 2020; Zhang, Dong, et al. 2022; Zhang, Li, et al. 2022; Zhao et al. 2024). Plant leaves have different adaptations under different environmental conditions of water, and they usually maintain narrow leaves to adapt to drought conditions and wide leaves to adapt to wet conditions (Zhang et al. 2020). In this study, under the 10‐day treatment at the beginning of the dry season, rain‐reduced *Fraxinus malacophylla* showed smaller leaf length and a lower specific leaf area, and rain‐increased *Fraxinus malacophylla* showed a larger leaf length and a higher specific leaf area. It was the same as the results of previous studies (Kursar et al. 2009; Markesteijn and Poorter 2009). This is the result of plants adapting to the external drought environment by reducing physiological activities and slowing down metabolism (Tomlinson et al. 2013). It indicates that in the dry season, *Fraxinus malacophylla* adapts to the environment with smaller leaves. In the dry season of this study, when the interval of rainfall was constant, the leaves mostly showed a larger leaf area and leaf shape index under natural rainfall. It might be because, in the dry season, *Fraxinus malacophylla* seedlings were rather more favorable to the growth of *Fraxinus malacophylla* seedlings under natural rainfall conditions because they were adapted to the moisture environment in central Yunnan. This is similar to the findings of Li (2010) on the leaf traits of *Nitraria tangutorum*, which showed that the leaf area, leaf perimeter, and leaf shape index of *Nitraria tangutorum* tended to increase when water was increased appropriately. It indicates that natural rainfall promotes the growth of *Fraxinus malacophylla* seedlings relative to rainfall reduction during the dry season. Leaf water content is an important trait of plant leaves, which can be used as a better predictor of plant photosynthesis and can reflect the ability of plants to produce nutrients (Wang, Fu, and Wang 2022). This also answers the question that needs to be solved in this study, that is, increasing water in the dry season can promote the growth of *Fraxinus malacophylla* seedlings. During the rainy season in this study, rain‐enhanced plants showed higher water content, and the leaf water content tended to be higher after prolonged rainfall intervals, while rain‐enhanced plants had a larger leaf perimeter and leaf area. This is because, during the rainy season, the leaves of *Fraxinus malacophylla* seedlings absorb more water, which promotes their photosynthesis to produce nutrients, which in turn promotes the growth of *Fraxinus malacophylla* seedling leaves. It is generally recognized that leaf trait variation in plants is an adaptive mechanism to cope with environmental changes (Halama and Reznick 2001) and plays an important role not only in plant response to short‐term changes in climate (e.g., rainfall) but also as a result of long‐term adaptive evolution, thus maintaining population survival and reproduction (Coleman, McConnaugyay, and Ackerly 1994). Leaf length, leaf width, leaf perimeter, and LMA of *Fraxinus malacophylla* seedlings were higher in the rainy season than in the dry season and were mostly optimal under natural rainfall, with an extended rainfall interval higher than the natural rainfall interval. The wider leaves absorbed a sufficient amount of water, promoted photosynthesis to create organic matter, accelerated seedling growth, and in turn adapted to this wet environment, forming an adaptive strategy for the rainy season. Overall, the leaves of *Fraxinus malacophylla* seedlings tended to grow better under natural rainfall and prolonged rainfall in the rainy season.

### 4.2 Leaf Photosynthesis Characteristics

Precipitation changes play an important role in global climate change, affecting ecosystem water balance, vegetation distribution, and physiological change processes of vegetation at different scales (Zhao, Wang, et al. 2023; Zhao, Wu, et al. 2023). It has been shown that both water deficit and excess can directly affect the performance and activity of plant photosynthesis, which in turn affects plant growth (Yi et al. 2022), and plant leaf Pn increases significantly after prolonged humidification (Wang, Li, et al. 2023; Wang, Zhang, et al. 2023). It has also been shown that the Pn, Gs, Ci, and Tr of plants first increase and then decrease with increasing water infusion (Li 2023). However, most studies have focused only on grassland ecosystems, while little has been done on the response of vegetation photosynthetic parameters to climate change in rocky desertification areas (Zheng et al. 2024). In this study, during the dry season, Pn, Gs, and Tr in the leaves of *Fraxinus malacophylla* seedlings showed an increasing and then decreasing trend with the increase of rainfall, and reached the peak under natural rainfall, which was the same as the previous study (Li 2023), indicating that a suitable water environment promotes photosynthesis of *Fraxinus malacophylla*, and that both rainfall increase and rainfall decrease inhibit its photosynthesis because the stomatal resistance of leaves increases under water stress, and the supply of CO_2_ to the chloroplasts is affected, resulting in a decrease in photosynthetic rate; however, Ci showed an increasing trend with the increase of rainfall under 5 days treatment, and a decreasing trend under 10 days treatment, which was again different from the previous study. supply was affected, resulting in a decrease in photosynthetic rate; however, Ci showed an increasing trend with increasing rainfall under the 5‐day treatment and a decreasing trend under the 10‐day treatment, which was again different from previous studies (Li 2023), that is, there was a phenomenon that Ci increased when Pn decreased in this study, which might be due to the fact that the decrease in Pn was not caused by the decrease in Gs. In the dry season of this study, both Gs and Tr were significantly lower in the reduced rainfall *Fraxinus malacophylla*, indicating that the stomatal opening of the leaves under water deficit was small, resulting in the leaf gas exchange process being affected, which in turn limited the CO_2_ supply and assimilation capacity, and consequently, the Pn and Ci, and also had an impact on the photosynthetic rate of the leaves, which was mainly caused by the stomatal factors; however, the increased rainfall and natural rainfall were relative to the extended rainfall, and the natural rainfall interval relative to extended rainfall interval, most of the leaf photosynthetic parameter indices remained high because water deficit was the limiting factor in these environments (Weiss et al. 2004; Kardol et al. 2010), this result that is consistent with the findings of Zhang et al. (2007). For WUE, a review of studies related to this study is shown in Table 3. Some researchers have found that WUE decreased with increasing precipitation by studying *Quercus ilex* (Ogaya and Peuelas 2003) in Europe. It has also been shown that plants maintain higher WUE under drought conditions to mitigate the effects of water deficits on plants as well as to improve the competition for water among plants under drought conditions. Reichstein et al. (2010) found that under extreme drought conditions, the photosynthetic rate of plants decreases with the decrease in soil water content, and the ecosystem WUE shows a tendency to decrease as well. Ning et al. (2018) also had a similar finding: WUE of irrigated and rainfed wheat showed a decreasing trend with decreasing precipitation. These previous findings are similar to most of the findings (Zhang, Wang, et al. 2023; Zhang, Yang, et al. 2023). However, the experimental results of Jiang and Dong (2000) in a sample zone in northeastern China showed that, with the increasing degree of drought, plant WUE showed a gradually increasing trend and began to decline after reaching a certain level, indicating that there is a certain threshold value of plant WUE in arid environments. In the dry season of this study, the WUE of 5 days showed a decreasing trend with the increase of rainfall, and the WUE of 10 days showed an increasing trend with the increase of rainfall, which is both the same and different from the previous study, the WUE of 5 days and 10 days showed the opposite trend of change with the increase of rainfall, it is because under the natural rainfall interval, the reduction of rainfall, although it makes the change of photosynthetic rate of the *Fraxinus malacophylla* is not obvious, the stomatal conductance was in a decreasing trend, which led to a decrease in transpiration rate, and then WUE was increased (Zou, Li, and Xu 2010), while prolonged rainfall interval, *Fraxinus malacophylla* needed to increase WUE to maintain its growth under the reduced frequency of rainfall. Another reason may be the uneven opening of plant stomata as a means of resisting drought and high temperatures to improve leaf transpiration and heat dissipation while maintaining a low WUE. These answers to the question that this study needs to solve, that is, natural rainfall interval and natural rainfall are more conducive to photosynthesis in the dry season and more suitable for the growth of *Fraxinus malacophylla* seedlings. In this study, at the beginning of the rainy season, Pn showed an increasing trend with increasing rainfall under the 5 days treatment, Ci was significantly higher under the 5 days treatment than under the 10 days treatment, while WUE was significantly higher under the 10 days treatment than under the 5 days treatment, which was similar to the results of Osonubi and Davies (2017), indicating that 5 days of rainfall increase at the beginning of the rainy season was favorable for the increase of photosynthesis rate of the *Fraxinus malacophylla* seedling, but prolonging the length of rainfall hindered the leaf CO_2_ uptake because the frequency of rainfall was reduced and plants closed their stomata in order to reduce transpiration, but because of the increase in rainfall, the increase in WUE in turn promoted the uptake of water by the leaves, which in turn increased their photosynthetic rate. During the rainy season of this study, Gs, Ci, and Tr basically all showed a decreasing trend with the increase of rainfall and reached the highest under the reduced rainfall, and the natural rainfall interval was higher than the prolonged rainfall interval, which indicated that the photosynthesis of *Fraxinus malacophylla* seedlings favored less rain under the natural rainfall interval during the rainy season, because during the rainy season in rocky desertification areas, less rain for many times means that it will not make the *Fraxinus malacophylla* rot due to flooding and flooding due to excessive moisture, nor will it affect its stomatal opening degree due to moisture deficit, thus affecting the photosynthesis. In the rainy season in the rocky desertification area, less rain will not cause the *Fraxinus malacophylla* to rot due to flooding, nor will it affect stomatal opening due to a water deficit, thus affecting photosynthesis. In this study, during the rainy season, the WUE was higher at 10 days than at 5 days. This is similar to previous findings that WUE in seasonal dry tropical forests decreases with increasing monthly rainfall and increases sharply with an increasing number of consecutive dry days (Costa et al. 2022), but there are examples of the opposite (Qiaoqi, Wayne, and Petra 2018). The reason for this is that the increase in air humidity and soil moisture content under the 5‐day treatment caused an increase in plant leaf Gs and Tr, which resulted in a decrease in water use efficiency. The experimental site of this study was located in central Yunnan. The *Fraxinus malacophylla* seedlings tended to promote photosynthesis under reduced rainfall in the rainy season, and extended rainfall intervals showed higher water use efficiency, which was attributed to the fact that *Fraxinus malacophylla* belongs to the drought‐ and barren‐tolerant plants, whose stomatal conductance is sensitive to changes in moisture. In conclusion, during the rainy season, plant leaves require less water and more frequent precipitation; that is, reduced rain and repeated rainfall promote the photosynthesis of *Fraxinus malacophylla*. These results are important for understanding the effects of climatic rainfall patterns on the photosynthetic growth of *Fraxinus malacophylla* in karst areas because *Fraxinus malacophylla* belongs to drought‐tolerant tree species, which play an important role in rocky desertification management, and understanding their response to rainfall patterns under climate change is of great value for afforestation in rocky desertification areas in southwest China.

**TABLE 3:** Retrieved WUE research reviews related to this study table.

Plant or forest type	Trends in different water environments	References
Plants of the Northeast China Sample Zone	Plant WUE increases and then decreases with increasing drought severity	Jiang and Dong (2000)
*Quercus ilex* under arid conditions	Decreases with increasing precipitation	Ogaya and Peuelas (2003)
*Phillyrea latifolia* under arid conditions	Maintaining high WUE under drought conditions	Ogaya and Peuelas (2003)
*Dieffenbachia Schott* in the Mediterranean under severe drought	Decreases with decreasing soil water content	Reichstein et al. (2010)
Irrigated and rainfed wheat	With increasing water stress, WUE decreased faster in dry‐crop wheat than in irrigated wheat	Ning et al. (2018)
Semiarid woodland ecosystems	During the summer months, WUE was higher in wet environments than in dry environments	Qiaoqi, Wayne, and Petra (2018)
Seasonal dry tropical forests	WUE decreased with increasing monthly rainfall and increased sharply with increasing number of consecutive dry days	Costa et al. (2022)
Semiarid grassland	Increased precipitation raises ecosystem WUE	Zhang, Wang, et al. (2023), Zhang, Yang, et al. (2023)
*Fraxinus malacophylla* during the dry season	WUE decreases with increasing rainfall as the frequency of rainfall decreases, and WUE increases with increasing rainfall as the frequency of rainfall increases	This study
*Fraxinus malacophylla* during the rainy season	WUE increases with longer rainfall intervals	This study

### 4.3 Leaf Fluorescence Characteristics

Chlorophyll fluorescence parameters reflect the “intrinsic” nature of plant photosynthesis and have been widely used in the study of photosynthesis in plants in adverse conditions (Liu, Jiang, and Yang 2012). The sunlight absorbed by plant chlorophyll is mainly used for chlorophyll fluorescence emission, heat dissipation, and photosynthetic electron transfer, which both cause changes in chlorophyll fluorescence parameters (Zhen and Shanguan 2006), and therefore chlorophyll fluorescence parameters are often used as an important indicator of the changes in plant stress tolerance under drought, flooding, salinity, and other stressful environmental conditions (Zhao, Wang, et al. 2023; Zhao, Wu, et al. 2023). Among them, PSII maximum photochemical quantum yield indicates the efficiency of PSII primary light energy conversion in plant leaves (Zhang et al. 2002), which indicates the magnitude of the ability of the PSII system to synthesize and utilize light energy. Fv/Fm is the maximum photochemical efficiency of PSII, whose value is constant under non‐stress conditions, at 0.80–0.83, and is independent of the species, but it will show a decreasing tendency under environmental stress conditions and even decrease sharply (Li, Dong, et al. 2017; Li, Tang, et al. 2017), but some studies have found no significant changes during stress (Liu et al. 2008; Komura et al. 2010). In this study, Fv/Fm was significantly higher at 5 days than at 10 days under the rain reduction treatment at the end of the dry season, and Fv/Fm was lower than 0.80 in all treatment groups, while in the rainy season, there was no significant difference between the Fv/Fm of the leaves of *Fraxinus malacophylla* seedlings under each treatment group, and the values of the Fv/Fm of the leaves of the *Fraxinus malacophylla* seedlings under each treatment group in the rainy season tended to be from 0.80 to 0.83, which indicated that the growth of the *Fraxinus malacophylla* was in an adversity environment at the end of the dry season, and in the rainy season, *Fraxinus malacophylla* was in a suitable environment for growth. This is similar to the results of the previous study (Wang, Fu, and Wang 2022), where *Deyeuxia angustifolia* showed a significant decrease in Fv/Fm under drought conditions, indicating that *Deyeuxia angustifolia* was photoinhibited under drought conditions to reduce the activity of the photosynthetic apparatus (Koichi et al. 2004). This also shows that water is essential for the growth and development of any plant. When rainfall is abundant, soil water content increases, and soil nutrient release and rate accelerate, which promotes nutrient cycling and thus photosynthesis in plants, and plant growth accelerates. In this study, the F0 under the treatment of 5 days at the beginning of the dry season showed a significant trend of decreasing and then increasing with the increase of rainfall, and the increase of the F0 value was mainly caused by the inactivation of some of the PSII reaction centers (Li, Dong, et al. 2017; Li, Tang, et al. 2017), which was the same as the results of the study by Shen (2021), and the initial fluorescence F0 of *Glycine max* also basically showed a trend of decreasing and then increasing, and the overall performance of drought control, this is an indication that the degree of destruction of PSII reaction centers in the leaves of *Glycine max* increased with the increase in water stress. Previous studies have shown that strong light, high temperatures, and a water deficit can cause photoinhibition (Ruban and Murchie 2012). The PI value reflects the ability and efficiency of photosynthesis in plants, and the higher the PI value, the stronger the photosynthetic capacity of the plant (Oukarroum, Schansker, and Strasser 2009). In this study, the PI was significantly higher at 5 days than at 10 days at the end of the dry season, and the overall PI value was higher under natural rainfall throughout the dry season. It indicates that the natural rainfall interval and natural rainfall amount are more favorable for the occurrence of photosynthesis in *Fraxinus malacophylla* in the dry season and more suitable for the growth of *Fraxinus malacophylla* seedlings. This may be because *Fraxinus malacophylla* itself is a drought‐ and barren‐tolerant species and the natural moisture conditions are already sufficient for its own growth and development process. The present study showed that prolonging the rainfall interval at the beginning of the rainy season significantly reduced the Fm of *Fraxinus malacophylla*, which is similar to the previous study (Saeid et al. 2016), and water deficit reduced the maximum fluorescence Fm of *Aloe vera* L. This is because *Fraxinus malacophylla* is a drought‐tolerant plant, and the reduction of rainfall frequency in the rainy season will affect the photosynthesis of *Fraxinus malacophylla* and inhibit growth. At the end of the rainy season, there was no significant difference in the fluorescence parameters of *Fraxinus malacophylla* leaves under each treatment group, probably because at the end of the rainy season, the soil water content, soil nutrients, air humidity, and so on had reached the optimal range for the growth of *Fraxinus malacophylla*, and at this time, changes in the amount of rainfall or changes in the interval of rainfall could not have any effect on the fluorescence characteristics of the leaves of *Fraxinus malacophylla*. It further shows that natural rainfall intervals and natural rainfall are more suitable for the growth of *Fraxinus malacophylla* seedlings during the dry season and that plant leaves require less water and more frequent water during the rainy season. Therefore, in the future dry and rainy seasons, the cultivation of live seedlings of *Fraxinus malacophylla* and the afforestation of *Fraxinus malacophylla* in rocky desertification areas can pay attention to the management of water.

### 4.4 Correlation Analysis and Principal Component Analysis

There is a close relationship between chlorophyll fluorescence and photosynthesis in leaves (Liu, Jiang, and Yang 2012). In the dry season in this study, Gs was closely and positively correlated with Tr in *Fraxinus malacophylla* seedlings, which is in agreement with the results of a previous study (Liu et al. 2022), which is because the stomata of *Fraxinus malacophylla* seedlings open to promote transpiration during the dry season when there is insufficient rainfall. In the dry season in this study, Pn was closely and positively correlated with Gs, which is the same as the results of Pourghayoumi, Bakhshi, and Rahemi (2017), and the photosynthetic rate and stomatal conductance of leaves were positively correlated. *Fraxinus malacophylla* has limited resistance to water adversity under drought conditions, with dysfunctional physiological and metabolic functions and reduced CO_2_ fixation by chloroplasts leading to non‐stomatal limiting factors, which make Pn lower (Wang et al. 2010). The results of this study showed that when *Fraxinus malacophylla* was under drought conditions, stomatal movement was restricted, Gs was reduced, and the plant's CO_2_ from the outside was greatly reduced, which led to a decrease in Ci. The stomata were close to completely closed during a severe water deficit, which resulted in a reduction in Tr and ultimately led to a reduction in plant Pn and a decrease in the photosynthetic capacity of the plant. In this study, Pn, Gs, and Tr were negatively correlated with F0 at the beginning of the dry season, which is consistent with the findings of Shen (2021) that Pn was negatively correlated with F0 under different moisture conditions in *Glycine max*. It is said that the degree of damage to the photosynthetic apparatus of the leaves of *Fraxinus malacophylla* seedlings was minimized under the treatment of increased rainfall, and the impact on photosynthetic electron transfer was minimized. With the decrease in rainfall, the degree of damage to the photosynthetic apparatus of the leaves was increasing, the impact on the photosynthetic electron transfer rate was increasing, and the impact on the net photosynthetic rate was increasingly large. In the rainy season of this study, Gs, Ci, and Tr were all closely negatively correlated with WUE, which is because the water use efficiency of *Fraxinus malacophylla* increases during the rainy season when there is more water, but the plant closes its stomata to avoid the dissipation of too much water through transpiration, and stomatal closure leads to a decrease in the uptake of CO_2_. In this study, there were positive correlations between leaf length and leaf width, or leaf width and leaf perimeter, or leaf length and leaf perimeter, in *Fraxinus malacophylla* seedlings during the dry and rainy seasons, which was the same finding by Chen (2015) that under different rain enhancement treatments, the leaf length, leaf width, leaf area, and leaf perimeter of the three functional groups of Poaceae, Fabaceae, and miscellaneous grasses most of them had significant positive correlations. It means that the leaf length and leaf width of *Fraxinus malacophylla* seedling leaves have little effect on changes in rainfall. In addition, leaf water content, Pn, Gs, Tr, WUE, and F0 of *Fraxinus malacophylla* can represent most of the information under different rainfall pattern treatments in the dry season, and leaf area, specific leaf mass, Ci, and Tr can represent most of the information in the rainy season. Therefore, in the future, research on the effect of rainfall patterns on the vegetation of rocky desertification areas can tend to focus on the measurement of these indexes, and at the same time, for the vegetation in rocky desertification areas, it is possible to increase or decrease the content of these indexes to do treatments to promote plant growth or reduce the content of these indicators to promote plant growth.

## 5 Conclusions

*Fraxinus malacophylla* has different adaptive responses to changes in rainfall patterns during the dry and rainy seasons. Increased water in the dry season promotes leaf growth, while increased water in the rainy season inhibits leaf growth. In the dry season, Pn, Gs, and Tr of *Fraxinus malacophylla* seedlings basically showed a tendency to increase and then decrease with the increase of rainfall and generally reached the highest level under the natural rainfall, and most of the leaf photosynthetic parameter indexes remained at a higher level with the increase of rainfall, natural rainfall relative to the decrease of rainfall, and the length of natural rainfall relative to the prolongation of the rainfall; throughout the dry season, the overall PI values were higher under the natural rainfall. In other words, the natural rainfall interval and the amount of natural rainfall were more conducive to the occurrence of photosynthesis in the dry season and more suitable for the growth of *Fraxinus malacophylla* seedlings. Extending the rainfall interval at the beginning of the rainy season significantly reduced the Fm of *Fraxinus malacophylla*, and the reduction of rainfall frequency in the rainy season affected the photosynthesis of *Fraxinus malacophylla* and inhibited the growth; throughout the rainy season, Gs, Ci, and Tr all tended to decrease with the increase of rainfall and reached the highest under the reduced rainfall, and the interval of natural rainfall was higher than the interval of the extended rainfall, and the leaves of the plant demanded less water and demanded water more times in the rainy season, that is, reduced rainfall and multiple rainfalls tended to promote photosynthesis in *Fraxinus malacophylla*. Correlation and principal component analysis of the indicators showed that there is a close relationship between chlorophyll fluorescence and photosynthesis in *Fraxinus malacophylla*; leaf water content, Pn, Gs, Tr, WUE, and F0 can be emphasized in the rainfall pattern of the dry season, and leaf area, LMA, Ci, and Tr can be emphasized in the rainy season. This study can provide a theoretical basis for the cultivation of live seedlings of *Fraxinus malacophylla*, afforestation, and vegetation restoration in karst, rocky desertification areas. However, the study was conducted in the experimental greenhouse and measured a portion of the physiological and biochemical indices of the *Fraxinus malacophylla*, which remained at the seedling level of the plant; in the future, it will be possible to measure some biomass and nutrient indicators and then transplant the *Fraxinus malacophylla* into the rocky desertification area, to monitor for a long period the dynamic changes in the growth of the *Fraxinus malacophylla* in the field in the rocky desertification area and its response to rainfall, and to further explore the soil structure and soil fertility suitable for the growth of the *Fraxinus malacophylla*, to provide better references for the restoration of the vegetation cover of the rocky desertification area under the background of the drastic changes in the global climate.

## Author Contributions

**Huiping Zeng:** conceptualization (lead), data curation (lead), formal analysis (lead), methodology (lead), resources (lead), software (lead), supervision (lead), validation (lead), writing – original draft (lead). **Xiaofei Cha:** conceptualization (lead), data curation (lead), formal analysis (lead), methodology (lead), resources (lead), software (lead), supervision (lead), validation (lead), writing – original draft (lead). **Lijuan Sun:** data curation (equal), methodology (equal), software (equal). **Huanxian Guo:** data curation (equal), methodology (equal), software (equal). **Shaojie Zheng:** data curation (equal), methodology (equal), software (equal). **Xingze Li:** data curation (equal), methodology (equal), software (equal). **Qiong Dong:** data curation (lead), funding acquisition (lead), methodology (lead), project administration (lead), resources (lead), supervision (lead), writing – review and editing (lead).

## Conflicts of Interest

The authors declare no conflicts of interest.

## Supporting information


Data S1.

## Data Availability

Data can be accessed via a public link for peer review at: http://osf.io/8cjt2/.
